# Differential diagnostic value of bilateral inferior Petrosal sinus sampling (BIPSS) in ACTH-dependent Cushing syndrome: a systematic review and Meta-analysis

**DOI:** 10.1186/s12902-020-00623-3

**Published:** 2020-09-17

**Authors:** Hao Wang, Ying Ba, Qian Xing, Run-Ce Cai

**Affiliations:** Department of Endocrinology, The First Affiliated Hospital of Dalian Medical University, Dalian, Liaoning Province China

**Keywords:** Bilateral inferior petrosal sinus sampling, ACTH-dependent Cushing syndrome, Differential diagnosis, Diagnostic meta-analysis

## Abstract

**Background:**

Previous studies have shown inconsistent results about the usefulness of bilateral inferior petrosal sinus sampling (BIPSS) in differential diagnosis of adrenocorticotropic hormone (ACTH)-dependent Cushing syndrome. This meta-analysis evaluated the diagnostic value of BIPSS via the published literature.

**Methods:**

This study searched PubMed, Embase, Web of Science, Cochrane library, and Wanfang database for published data on the use of BIPSS in Cushing syndrome differential diagnosis as of October 2019. Sensitivity, specificity, positive likelihood ratio (PLR), negative likelihood ratio (NLR), diagnostic odds ratio (DOR), and receiver operating characteristic (ROC) curves were calculated based on the relevant data.

**Results:**

This meta-analysis included a total of 23 studies with 1642 patients. The calculated sensitivity, specificity, PLR, and NLR were 0.94 (95% confidence interval, CI: 0.91–0.96), 0.89 (95% CI: 0.79–0.95), 8.8 (95% CI: 4.3–17.9), and 0.07 (95% CI: 0.04–0.11), respectively. The pooled DOR and area under the ROC curve were 129 (95% CI: 48–345) and 0.97 (95% CI: 0.95–0.98), respectively.

**Conclusion:**

This meta-analysis indicated that BIPSS had high diagnostic value for detecting ACTH in patients with ACTH-dependent Cushing syndrome, and BIPSS should be used as an effective method to identify ACTH-secretion sources.

## Background

Adrenocorticotropic hormone (ACTH)-dependent Cushing syndrome (CS) is caused by excessive secretion of ACTH by the pituitary or pituitary tumors, causing bilateral adrenal hyperplasia and excessive cortisol secretion with clinical manifestations such as a moon-shaped face, buffalo hump, and hypertension. The majority of ACTH-dependent Cushing syndrome cases are caused by Cushing disease (CD), a condition in which ACTH-secreting tumors are responsible for elevated ACTH levels. Other cases, such as ectopic ACTH syndrome (EAS), have ectopic sources. These have different therapeutic principles and prognoses. Based only on clinical manifestations, detection of cortisol levels and ACTH, high- and low-dose dexamethasone suppression tests, and imaging, these conditions are not completely distinguishable. Studies have shown that non-functional pituitary tumors are common [1–3], suggesting that even if a pituitary tumor is revealed by magnetic resonance imaging (MRI), the tumor is not necessarily the source of the ACTH. Some ACTH-secreting tumors are small in size, and may not be revealed by MRI. Only 50–70% of these tumors are diagnosed [4, 5]. Therefore, negative MRI does not completely exclude ACTH-secreting tumors. In high-dose dexamethasone suppression test (HDDST), most ACTH-secreting tumors are suppressed, while most EASs are unrepressed. However, a small number of patients have unpredicted presentations on HDDST [6, 7]. An HDDST cannot effectively distinguish between ACTH-secreting tumors and EAS. Therefore, the localization rate of ACTH-secreting tumors is very low. In addition to the positive rate of MRI detection mentioned above, the HDDST has approximately 78–81% sensitivity and 67–81% specificity [8, 9],while the corticotrophin-releasing hormone (CRH) stimulation test has 76–91% sensitivity and 95% specificity [10, 11]. For these reasons, more effective diagnostic approaches are needed to distinguish the two diseases.

Bilateral inferior petrosal sinus sampling (BIPSS) has been considered to be the gold standard for differential diagnosis of the above two diseases. BIPSS is an interventional method in which a blood sample from the bilateral inferior petrosal sinus and a peripheral blood sample are used to measure ACTH by calculating the lower sinus/peripheral (IPS/P) ACTH ratio and left and right inferior petrosal sinus (IPS/IPS) ACTH ratio. The IPS/P ACTH ratio is used to distinguish between CD and EAS. In general, an IPS/P ACTH ratio of ≥2 before a CRH stimulation test and an IPS/P ACTH ratio of ≥3 after the CRH test are criteria for diagnosing CD [6]. These diagnostic criteria are also recommended by other centers [12, 13]. The ratio of ACTH between the left and right IPS is used to determine the location of pituitary microadenomas, with IPS/IPS > 1.4 indicating a tumor located at the side with higher ACTH, and IPS/IPS ≤ 1.4 indicating a tumor locating near the midline [6]. Studies have shown that vasopressin receptor is present on the surface of ACTH-secreting tumors, and administration of vasopressin stimulates the release of ACTH [14]. Application of desmopressin (DDAVP) during BIPSS enhances diagnostic accuracy [15]. Generally speaking, although BIPSS is a mildly invasive examination, it is relatively safe. It has occasional complications, including groin hematoma, cerebral hemorrhage, and vasovagal reactions (VVRs) [12, 16, 17]. The incidence of groin hematoma is approximately 4%, and the incidences of cerebral hemorrhage and vasovagal reactions (VVRs) are below 1%. Occasional pulmonary embolism is also reported by some researchers. However, meta-analysis of BIPSS is currently unavailable. This study performed a meta-analysis of BIPSS for the differential diagnosis of ACTH-dependent Cushing syndrome and evaluated the differential diagnostic value for this condition.

## Methods

### Literature search

This study strictly followed the Preferred Reporting Items for Systematic reviews and Meta- Analyses (PRISMA) guidelines [18] and used PubMed, Embase, Web of Science, Cochrane Library, and Wanfang databases to search for studies using BIPSS for the differential diagnosis of ACTH-dependent Cushing syndrome as of October 2019. The following search terms were used: petrosal sinus sampling, bilateral inferior petrosal sinus sampling, Cushing’s syndrome, Cushing disease, and ectopic Cushing syndrome. The search strategies in the various databases were as follows: PubMed: (“petrosal sinus sampling” [Mesh]) AND “Cushing’s syndrome” [Mesh]); Embase: (Emtree term-exploded = Cushing’s syndrome AND Abstract = petrosal sinus sampling); Web of Science: TS = (petrosal sinus sampling AND Cushing’s syndrome); and Cochrane Library and WanFang: keyword = (petrosal sinus sampling AND Cushing’s syndrome). During searching, keywords and free words were used simultaneously. Manual searches were also used, and relevant references included in the extracted papers were also searched. Literature was searched by two of the authors (Hao Wang, Run Ce-Cai) independently.

### Inclusion and exclusion criteria

The inclusion criteria of this meta-analysis were as follows: (1) patients confirmed with Cushing syndrome (CS) and unclear ACTH source; (2) CS caused by ACTH-secreting tumor or EAS confirmed by postoperative pathology or by clinical manifestations, biochemical tests, and surgery; (3) the study provided true positives (TP), false positives (FP), false negatives (FN), and true negatives (TN) or the data for the calculation of TP, FP, FN, and TN. The exclusion criteria were: (1) studies with incomplete data or data which could not be used to calculate the contingency table, (2) non-original studies, (3) repeated studies, (4) animal studies, and (5) studies with less than 20 patients included.

### Data extraction

Two authors (Qian Xing, Ying Ba) read the included papers and extracted relevant data through discussion. In case of disagreement, another author (Hao Wang) was involved in further discussion. Contents of data extraction in the literature included: name of the first author, year of publication, country of the study, study design (prospective and retrospective), the application of CRH or DDAVP stimulation, the application of prolactin (PRL) correction, TP, FP, FN, and TN.

### Quality assessment

The quality of the included studies was evaluated by two (Qian Xing, Ying Ba) of the authors independently using the Quality Assessment of Diagnostic Accuracy Studies (QUADAS-2) [19, 20] according to the four aspects as follows: selection of cases, trials to be assessed, gold standard, and flowchart and progress of cases. Each of the assessments contained seven items which were answered as “yes,” “no,” or “uncertain.” An answer of “yes” indicated that the risk offset of the study was low, while the answers of “no” and “uncertainty” indicated high risk offset.

### Data synthesis and analysis

We used a bivariate model proposed by Reitsma et al. [21] for the meta-analysis of the included studies, which was performed using the MIDAS module of STATA version 14.0. Sensitivity, specificity, positive likelihood ratio (PLR) and negative likelihood ratio (NLR) and the 95% confidence intervals (95%CIs) were calculated [22]. The sensitivity and specificity of each included study were used to plot the summary receiver operator characteristic (SROC) curve and calculate the area under the SROC curve (AUC) [23]. The AUC can be statistically interpreted as the probability to correctly distinguish patients from normal controls. The I^2^ test was conducted to analyze the heterogeneity between studies, which I^2^ more than 50% indicated that there is a substantial between-study heterogeneity. A meta-regression analysis of the diagnostic odds ratio (DOR) was performed according to the study design, year of publication, country of publication, application of CRH or DDAVP, application of PRL correction, and the number of patients included in the study [24, 25]. Deek’s asymmetry test was used to evaluate whether a publication bias existed [26].

## Results

**Fig. 1:**
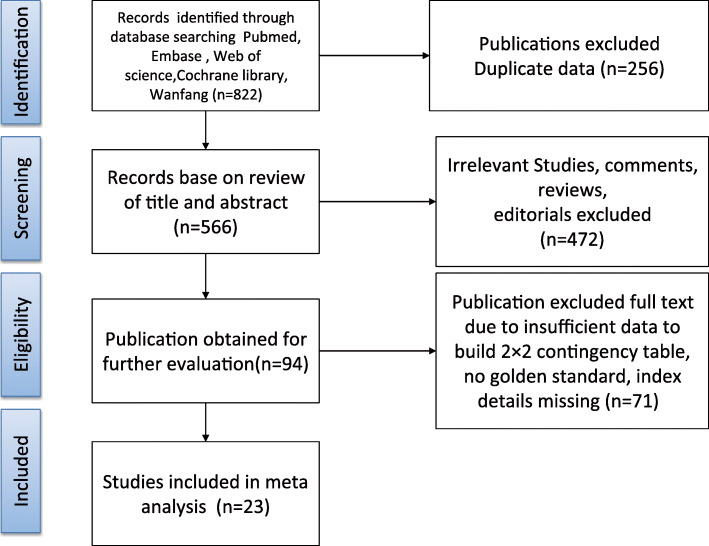
Retrieval flowchart to obtain study data for meta-analysis

As shown in Fig. 1 which describes the literature searches and the workflow for study inclusion, there were 822 articles in the initial search, but 256 of them were found to be duplicated and were removed from further analysis. In addition, a total of 472 articles included irrelevant research articles, reviews, commentaries, editorials, and letters, which were further removed. Of the remaining 94 articles, those that contained incomplete data, replicated research, no gold standard, incomplete research descriptions, or less than 20 patients were also removed. Thus, a total of 23 studies were included in this meta-analysis [3, 6, 12, 13, 27–45].

**Fig. 2:**
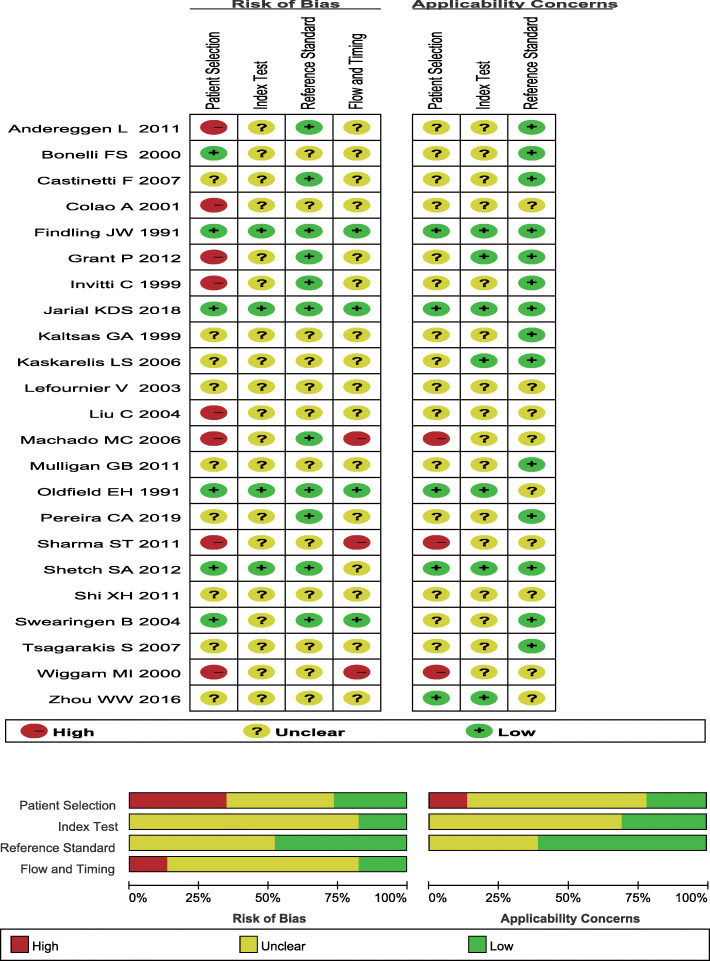
Quality assessments for the included studies

Table 1 shows the characteristics of the included studies which were published in 1991–2019, including 11 studies conducted in Europe, nine studies conducted in the United States or Brazil, and 3 studies conducted in China or India. There were 3 prospective studies and 20 retrospective studies included in this meta-analysis. Figure 2 shows the quality of the included studies.

**Table 1:** Characteristics of the included studies

Author	Year	Country	Design	Stimulation	PRL adjust	Gold Standard	TP	FP	FN	TN
Oldfield EH [6]	1991	USA	pro	CRH	No	Pathology	203	0	0	17
Findling JW [27]	1991	USA	pro	CRH	No	Pathology	18	3	2	6
Kaltsas GA [28]	1999	UK	retro	CRH	No	Pathology	50	0	19	6
Invitti C [29]	1999	Italy	retro	DDAVP	No	Pathology	65	0	11	9
Bonelli FS [13]	2000	USA	retro	CRH	No	Pathology	71	1	6	9
Wiggam MI [30]	2000	Northen Ireland	retro	CRH	No	Pathology	36	0	8	1
Colao A [12]	2001	Italy	retro	CRH	No	Pathology	60	0	8	10
Lefournier V [31]	2003	France	retro	CRH	No	Pathology	65	2	4	6
Swearingen B [32]	2004	USA	retro	CRH	Yes	Pathology	70	2	9	2
Liu C [33]	2004	USA	retro	CRH	No	Pathology	39	0	3	9
Kaskarelis LS [3]	2006	Greece	retro	CRH	No	Pathology	40	3	6	5
Machado MC [34]	2006	Brazil	retro	CRH	No	Pathology	46	0	1	5
Castinetti F [35]	2007	France	retro	DDAVP	Yes	Pathology	32	0	4	7
Tsagarakis S [36]	2007	Greece	retro	CRH	No	Pathology	46	0	1	7
Shi XH [37]	2011	China	retro	No	No	Pathology	58	1	10	4
Mulligan GB [38]	2011	USA	retro	CRH	No	Pathology	33	1	2	1
Andereggen L [39]	2011	Switzerland	retro	CRH	No	Pathology	19	1	1	2
Sharma ST [40]	2011	USA	retro	No	No	Pathology	16	1	1	7
Shetch SA [41]	2012	USA	retro	CRH	Yes	Pathology	195	5	12	5
Grant P [42]	2012	UK	retro	DDAVP	No	Pathology	72	1	0	10
Zhou WW [43]	2016	China	pro	No	No	Pathology	84	1	3	5
Jarial KDS [44]	2018	India	pro	CRH	No	Pathology	26	0	0	2
Pereria CA [45]	2019	Portugal	retro	No	No	Pathology	27	0	1	2

**Fig. 3:**
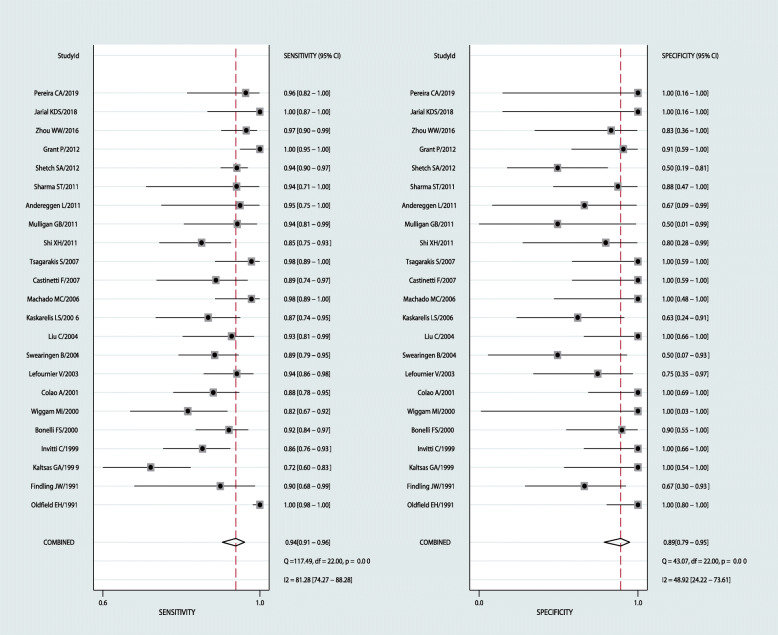
Forest plot for sensitivity and specificity

**Fig. 4:**
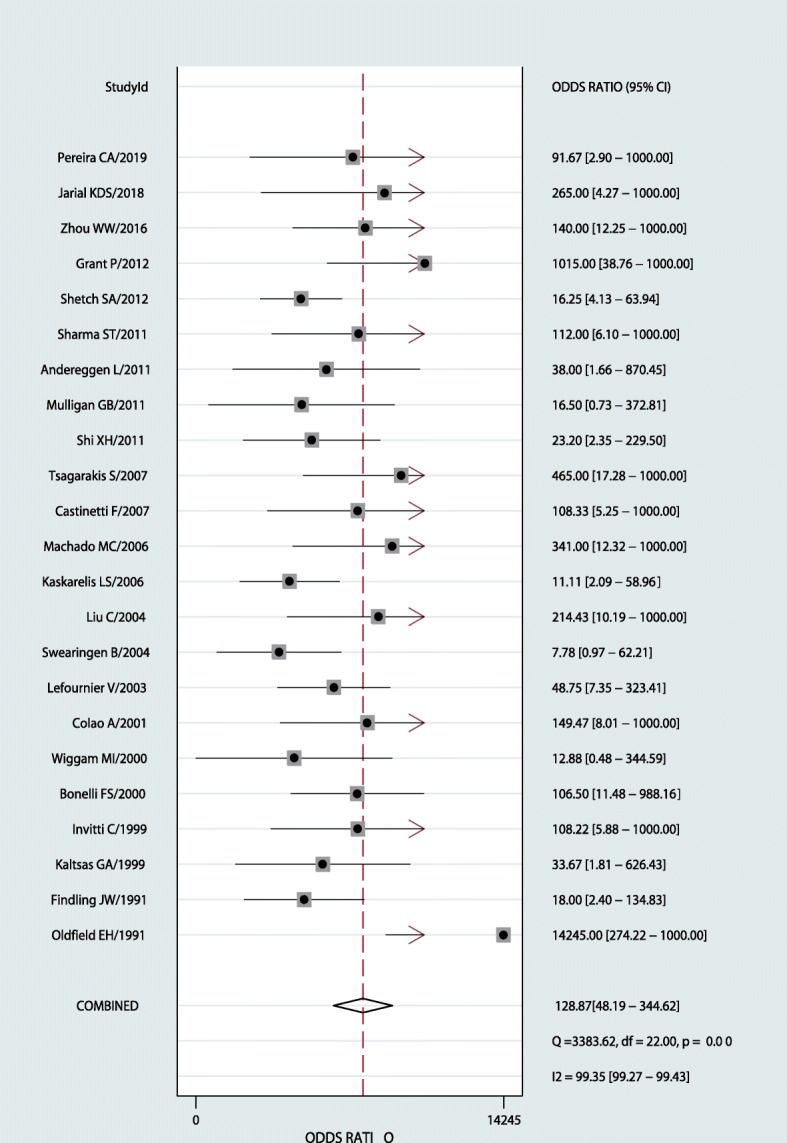
Forest plot for DOR

**Fig. 5:**
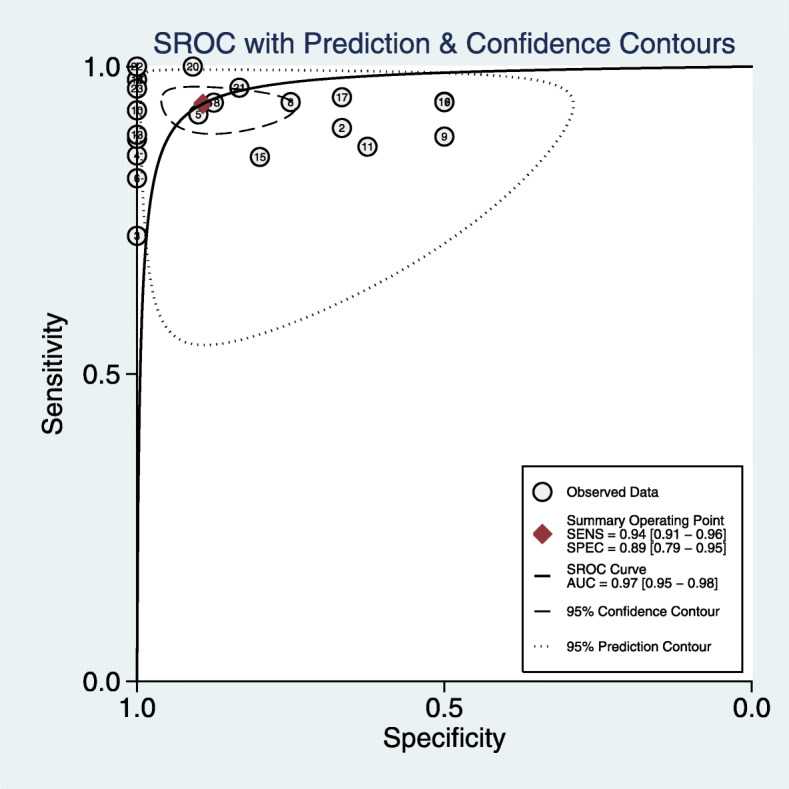
Area under the ROC curve

Results were given as values (95%CI). Using a random-effect model, the results were as follows: sensitivity 0.94 (0.91–0.96), specificity 0.89 (0.79–0.95; Fig. 3), PLR 8.8 (4.3–17.9), and NLR 0.07 (0.04–0.11). DOR 129 (48–345; Fig. 4), *P* = 0.00,I^2^ = 99.35%; AUC 0.97 (0.95–0.98; Fig. 5).

**Fig. 6:**
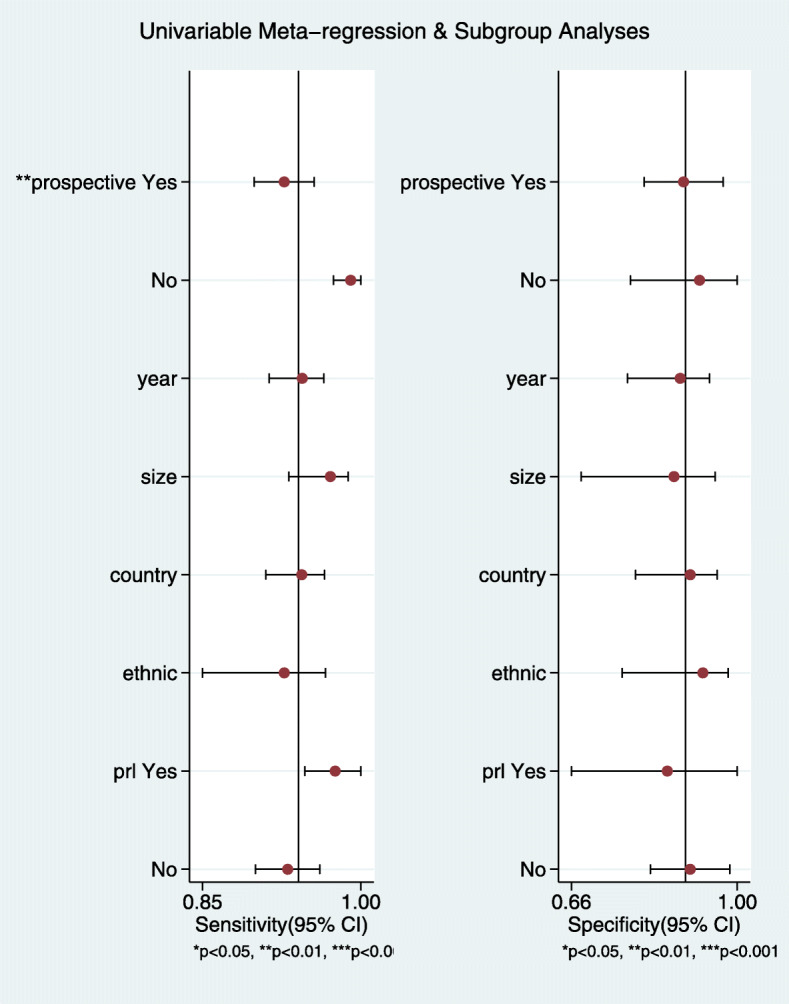
Meta-regression analysis for DOR

**Fig. 7:**
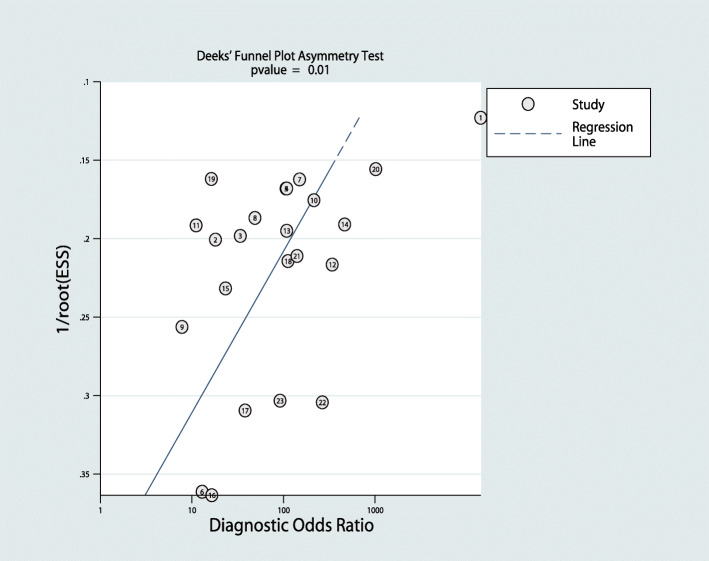
Deek’s plot for BIPSS in the differential diagnosis of ACTH-dependent CS

A meta-regression analysis was performed based on the study design (prospective or retrospective), year of publication, country of publication, sample size (number of patients enrolled being 21–100,100–200,>200), patient ethnicity, application of CRH or DDAVP, and application of PRL correction (Fig. 6). The results suggested that the research design was the main cause of heterogeneity. Deek’s asymmetry test was used to detect the presence of publication bias, and the results indicated a publication bias (*P* = 0.01; Fig. 7).

## Discussion

This study was the first meta-analysis to evaluate the differential diagnostic value of BIPSS in ACTH-dependent Cushing syndrome. It included a total of 23 studies and 1642 patients. Our results suggested that the sensitivity and specificity of BIPSS to pituitary or ectopic ACTH were 94 and 89%, respectively, indicating that BIPSS has high value in the differential diagnosis of ACTH-dependent Cushing syndrome. In addition, the DOR value was also high, suggesting that BIPSS could effectively identify the ACTH source. The area under the SROC curve was 0.97, suggesting that the overall diagnostic performance of BIPSS was effective.

BIPSS has a high value in the differential diagnosis of CS cases that have typical CS presentations clinically and biochemically but have an unclear ACTH source. Because BIPSS does not identify the ACTH source from a morphological perspective, but from a functional perspective, this diagnostic approach is accurate, with relatively high sensitivity and specificity. CD accounts for a large proportion of ACTH-dependent Cushing syndrome cases, and BIPSS is particularly suitable for patients with negative MRI results. Furthermore, BIPSS provides an important basis for guiding the surgical treatment of this disease.

In most cases, the ACTH level of CD was lower than EAS. For example, the ACTH level of the ACTH-secreting tumors was 111.35 pg/ml, while the ACTH level of EAS was 277.01 pg/ml [37]. After CRH or DDAVP stimulation, the ACTH level increased significantly. Many researchers believe that the stimulation intensity of DDVAP on ACTH-secreting tumors is weaker than that of CRH. For example, in Jarial’s study, the ACTH (IPS/P) ratio of ACTH-secreting tumors is increased 11.6-fold after DDAVP stimulation. After CRH stimulation, the ratio is increased by 28-fold [44]. In terms of the maximum ACTH level after stimulation, Bonelli’s study showed that the ACTH levels reached 1062 pg/ml after DDAVP stimulation, and 3058 pg/ml after CRH stimulation [13]. This leads naturally to the question of why the stimulation intensity of DDAVP is weaker than that of CRH. We believe that CRH directly stimulates ACTH, and stimulation of DDVAP is due to the presence of vasopressin receptor. Thus, the stimulation intensity of DDVAP is weaker than that of CRH.

BIPSS has a high differential diagnostic value for CD and EAS. Application of CRH or DDVAP stimulation enhances the sensitivity and specificity of BIPSS. However, BIPSS should still be combined with other diagnostic methods, such as imaging, HDDST, and the low-dose dexamethasone suppression test for comprehensive diagnosis.

False negative results can occur in BIPSS. These have been reported to be approximately 10% [32], and may be related to operational failure or abnormal venous drainage from the inferior petrosal sinus. BIPSS is not ideal for identifying the diseased side [31, 46], which may be due to the presence of branches joined to the cavernous sinus and frequent contralateral venous return. A previous study used cavernous sinus sampling instead of BIPSS to obtain a good differential diagnosis for CD and EAS [47]. For BIPSS, the success rate is closely related to the operator’s technique and experience, and accurate catheterization is very important. Results of a previous study suggest that PRL for correction improves the success rate of catheterization [48].

An interesting consideration is whether the false positive rate of BIPSS increases among the patients with positive MRI results, which is only discussed in few studies. The study by Kaskarelis et al. showed that 1 out of 23 MRI-positive patients had a BIPSS-false-negative result (4.3%) and 2 out of 55 MRI-negative patients had BIPSS-false-negative results (3.6%) [3], while the majority of BIPSS-false-positive rates in CS patients ranged from 0 to 5%. Thus, the BIPSS-false-positive rate of the MRI-positive patients in Kaskarelis et al.’s study was higher than that of the MRI-negative patients, and also higher than the average of most other studies. It may be related to the change of ACTH releasing mediated by CRH / vasopressin receptor. Since few studies were related to this issue, further studies with increased sample sizes are needed for verification. Another interesting issue should be mentioned that, furthermore, corticotroph hyperplasia must be considered a possibility in MRI-negative false positive BIPSS as hyperplasia often mimics adenoma biochemically but will show high variability on pathology.

This meta-analysis provided implications for future studies as follows: PRL can be used as a reference to improve the accuracy of catheterization during BIPSS. CRH or DDAVP stimulation should also be used during BIPSS to improve the sensitivity and specificity.

The study had several strengths. To begin with this was the first meta analysis to use as many as 1642 cases to summarize the diagnostic value of BIPSS in ACTH-Dependent Cushing Syndrome, which gave improved statistical power to the findings. Moreover, we excluded studies with less than 20 patients included, which means our data came from more reliable centers and more experienced doctors. Thirdly, the bivariate model uses pairs of sensitivity and specificity as the starting point of the analysis and thus may be more reliable for estimating the diagnostic accuracy. Finally, meta-regression analysis suggested that the experimental design can explain the source of heterogeneity.

However, our meta-analysis also had some limitations. First, we did not include grey literature, but only published studies which might cause a selection bias, and the publication bias of this meta-analysis was *P* < 0.05, suggesting the presence of publication bias. The possible reasons for this were that (1) BIPSS had high diagnostic accuracy of TP and TN for determining the ACTH source and likely shows the ideal statistical results in the software, leading to the calculation of publication bias; (2) authors might have submitted studies only with positive results to increase the chance of being published; and (3) this meta-analysis only included studies published in Chinese and English. Furthermore, many of the included studies were retrospective studies, and the integrity and homogeneity of the data were not guaranteed, which may had adverse effects on the research results.

This study was the first meta-analysis to evaluate BIPSS’s effects on determining the etiology of ACTH-dependent Cushing syndrome, suggesting that BIPSS had a great differential diagnostic value for the ACTH source. Results of this study require further large-scale prospective studies to validate the differential diagnostic value of BIPSS for ACTH-secretion sources in different patients.

## Conclusion

This meta-analysis indicated that BIPSS had a high diagnostic value for patients with ACTH-dependent Cushing syndrome, and as such, BIPSS should be used as an effective method to identify ACTH-secretion sources. CRH or DDAVP stimulation should be used during BIPSS to improve the test’s sensitivity and specificity.

## Supplementary information

**Additional file 1.** PRISMA checklist.

## Data Availability

Not applicable. This study is a systematic review and we used primary data, which are already publicly available. The datasets used and/or analysed during the current study are available from the corresponding author on reasonable request.

## Abbreviations

BIPSS: Bilateral inferior petrosal sinus sampling
ACTH: Adrenocorticotropic hormone
CS: Cushing syndrome
CD: Cushing disease
EAS: Ectopic ACTH syndrome
HDDST: High-dose dexamethasone suppression test
CRH: Corticotrophin- releasing hormone
